# Multiple DNA Binding Proteins Contribute to Timing of Chromosome Replication in *E. coli*

**DOI:** 10.3389/fmolb.2016.00029

**Published:** 2016-06-28

**Authors:** Leise Riber, Jakob Frimodt-Møller, Godefroid Charbon, Anders Løbner-Olesen

**Affiliations:** Section for Functional Genomics and Center for Bacterial Stress Response and Persistence, Department of Biology, University of CopenhagenCopenhagen, Denmark

**Keywords:** *E. coli*, chromosome replication, DNA binding proteins, cell mass, initiation synchrony

## Abstract

Chromosome replication in *Escherichia coli* is initiated from a single origin, *oriC*. Initiation involves a number of DNA binding proteins, but only DnaA is essential and specific for the initiation process. DnaA is an AAA+ protein that binds both ATP and ADP with similar high affinities. DnaA associated with either ATP or ADP binds to a set of strong DnaA binding sites in *oriC*, whereas only DnaA^ATP^ is capable of binding additional and weaker sites to promote initiation. Additional DNA binding proteins act to ensure that initiation occurs timely by affecting either the cellular mass at which DNA replication is initiated, or the time window in which all origins present in a single cell are initiated, i.e. initiation synchrony, or both. Overall, these DNA binding proteins modulate the initiation frequency from *oriC* by: (i) binding directly to *oriC* to affect DnaA binding, (ii) altering the DNA topology in or around *oriC*, (iii) altering the nucleotide bound status of DnaA by interacting with non-coding chromosomal sequences, distant from *oriC*, that are important for DnaA activity. Thus, although DnaA is the key protein for initiation of replication, other DNA-binding proteins act not only on *oriC* for modulation of its activity but also at additional regulatory sites to control the nucleotide bound status of DnaA. Here we review the contribution of key DNA binding proteins to the tight regulation of chromosome replication in *E. coli* cells.

## Timing of initiation of chromosome replication in *E. coli*

**Figure 1:**
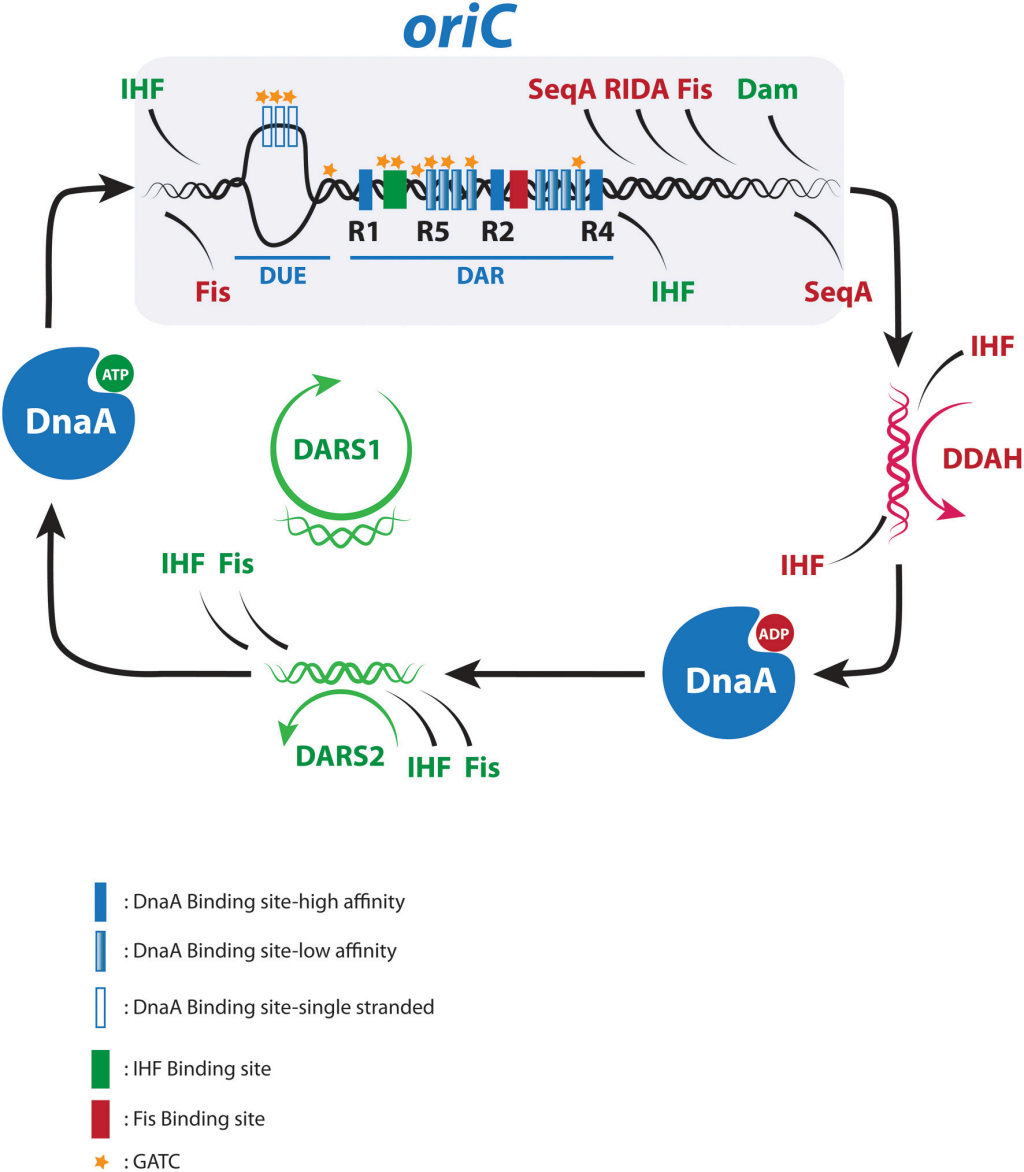
**The chromosome replication cycle**. Dynamic binding of activators (green) and inhibitors (red) to *oriC* and distal regulatory sequences during the replication cycle. For details see text.

Chromosome replication in *Escherichia coli* is initiated from a single replication origin, *oriC*. The *oriC*-encoded structural and functional instructions for initiation are well-described (Leonard and Mechali, 2013; Skarstad and Katayama, 2013). In brief, the minimal *oriC* contains two functional regions: the Duplex Unwinding Element (DUE), which comprises three AT-rich repeat sequences of each 13 bp, and the flanking DnaA Assembly Region (DAR) (Figure 1; Mott and Berger, 2007; Ozaki and Katayama, 2012). DnaA is the initiator protein responsible for DUE opening and for the recruitment of replisome components and is the only protein that is both essential and specific for the initiation process (Kaguni, 2011; Leonard and Grimwade, 2011). DnaA belongs to the AAA+ proteins (ATPases Associated with diverse Activities) and can bind both ATP and ADP with similar high affinities (Sekimizu et al., 1987). The DAR region contains high affinity DnaA Boxes (R1, R4, and R2) that bind both DnaA^ATP^ and DnaA^ADP^, along with multiple low affinity sites (R3, R5/M, I1, I2, I3, C1, C2, C3, τ1, and τ2) that bind DnaA^ATP^ (McGarry et al., 2004; Kawakami et al., 2005; Rozgaja et al., 2011). The DAR region also contains recognition sequences for two additional DNA binding proteins*;* IHF and Fis (Figure 1; Polaczek, 1990; Gille et al., 1991).

Throughout most of the cell cycle *oriC* is bound by DnaA located at R1, R2, and R4. This origin recognition complex (ORC) serves dual purposes in setting the stage for proper orisome assembly and preventing premature DNA unwinding. The ratio of DnaA^ATP^ to DnaA^ADP^ varies through the cell cycle and the peak at about 70–80% DnaA^ATP^ coincides with replication initiation (Kurokawa et al., 1999). In the current model for orisome formation, two converging DnaA^ATP^ filaments are formed (Rozgaja et al., 2011). One filament originates from R4 and grows leftward. This R4-filament displaces Fis from its binding site next to R2, which allows IHF to bind its recognition sequence next to R1. IHF bends the DNA 180° thereby bringing R1 in proximity of R5 and allows for the formation of the rightward filament responsible for duplex opening at the DUE, DnaC assisted helicase loading and assembly of the replisome (Leonard and Grimwade, 2011, 2015; Ozaki et al., 2012). Following initiation, DnaA^ATP^ is converted to DnaA^ADP^ primarily by a process called regulatory inactivation of DnaA (RIDA), which is dependent on the Hda protein bound to ADP and the DNA-loaded β-clamp of the polymerase III holoenzyme (Kato and Katayama, 2001), and by the less efficient *datA*-dependent DnaA^ATP^ hydrolysis (DDAH). DDAH takes place at *datA* and is dependent on IHF (Figure 1; Kasho and Katayama, 2013).

### Coordination of initiations with cell mass increase

A long standing observation is that initiation of chromosome replication occurs when a certain cellular mass per origin, the initiation mass, is reached (Donachie, 1968; Hill et al., 2012). This coupling of replication initiation to cell growth depends on the DnaA protein. Earlier studies indicate that accumulation of DnaA protein sets the time of initiation in the cell cycle especially around or below wild-type level (Løbner-Olesen et al., 1989). On the other hand, a coordinated increase in DnaA^ATP^ and DnaA^ADP^ does not significantly increase initiation (Kurokawa et al., 1999; Flatten et al., 2015), suggesting that accumulation of DnaA^ATP^ is insufficient to trigger initiation. However, in the absence of RIDA, where DnaA is mainly ATP bound, a modest increase in DnaA^ATP^ level leads to excessive initiations from *oriC* (Riber et al., 2006; Fujimitsu et al., 2008), as does expression of a DnaA mutant protein insensitive to RIDA (Simmons et al., 2004). Together, this indeed suggests that accumulation of DnaA^ATP^ triggers initiation, whereas this effect can be offset by a similar increase in DnaA^ADP^ (Donachie and Blakely, 2003). The participation of DnaA^ADP^ in orisome formation remains unclear (Leonard and Grimwade, 2015), but the above observations suggest that it affects initiation negatively. Overall, accumulation of DnaA protein during steady-state growth, along with the cell cycle specific peak in DnaA^ATP^/DnaA^ADP^ratio, determines the onset of initiation with little variation between individual cells.

### Coordination of initiations within a single cell

**Figure 2:**
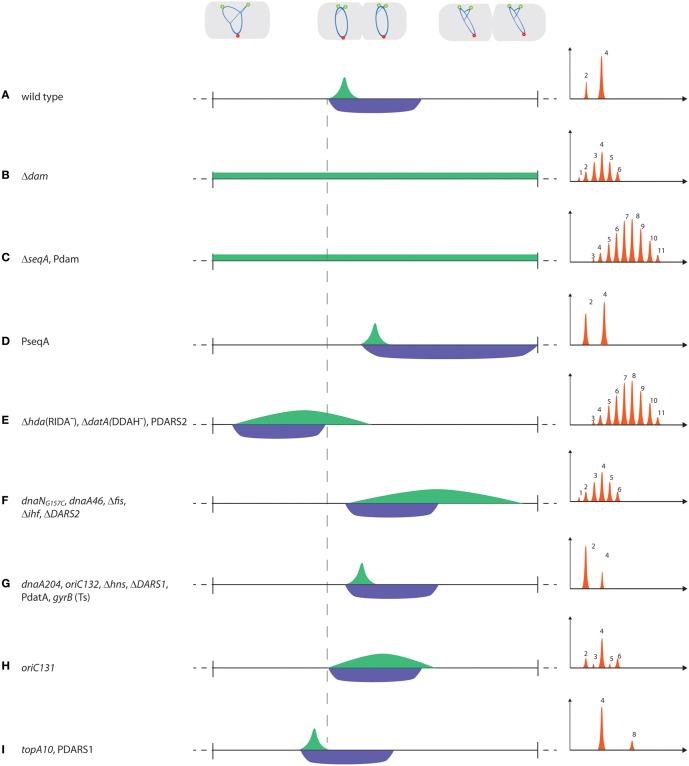
**Timing of replication initiation**. Examples of mutants/plasmids with altered initiation (I^P^; green) and sequestration (S^P^; blue) periods. The horizontal line represents one doubling time, whereas the vertical (hyphenated) line illustrates the time of initiation of the first origin in wild-type cells. Note that the start of S^P^ always coincides with the first origin initiated, i.e., start of I^P^. In the graphical representation of initiation synchrony, the number of origins per cell are on the X-axis, whereas the cell number is on the Y-axis of each histogram. When more than one mutation/plasmid is listed for a specific example (e.g., in C,E–G,I), the histograms are representative of the initiation phenotype of each individual mutation/plasmid.

In individual cells, initiation at all origins occurs within approximately 1/10 of the doubling time (Initiation period, I^P^; Figure 2A). Rapidly growing cells with overlapping replication cycles therefore predominantly contain 2^n^ (*n* = 1, 2, 3) copies of *oriC*, referred to as initiation synchrony (Skarstad et al., 1986). Initiation synchrony depends on the immediate inactivation of newly replicated origins by sequestration. *oriC* contain 11 copies of the sequence GATC that are methylated by Dam methyltransferase and bound, i.e., sequestered, by SeqA when hemimethylated. Sequestration prevents DnaA binding to its weak sites in *oriC* (Nievera et al., 2006) for approximately 1/3 generation (Sequestration period, S^*P*^; Figure 2A) and serves to keep track of which origins have been initiated (Boye and Løbner-Olesen, 1990; Campbell and Kleckner, 1990; Lu et al., 1994). The ability to initiate all origins in synchrony could result from maintaining a high DnaA^ATP^ level throughout I^P^. Alternatively the first origin initiated may release its DnaA^ATP^ to assist in triggering successive initiations at remaining origins in a cascade-like manner to ensure that free DnaA^ATP^ increases through I^P^ and enforces synchrony (Løbner-Olesen et al., 1994). These models predict different outcomes for sequestration deficient cells. A high DnaA^ATP^ level throughout I^P^ would result in re-initiation(s) within I^P^, asynchrony and overinitiation. The cascade model predicts a delay between successive initiations due to newly initiated origins competing with old origins for a limited amount of DnaA^ATP^. The initiation frequency would be directly proportional with accumulation of DnaA^*ATP*^ resulting in asynchrony but an unchanged overall initiation frequency, which is in accordance with experimental observations for Dam deficient cells (Boye and Løbner-Olesen, 1990; Løbner-Olesen et al., 1994).

Synchrony is only observed when I^P^ < S^P^ (Figure 2A). In cells with aberrant timing of initiation, the I^P^ and S^P^ periods change, i.e., either start earlier in the cell cycle at a decreased initiation mass, i.e., overinitiation, or are delayed with an increased initiation mass, i.e., underinitiation. Alternatively, the duration of I^P^ and S^P^ may change relative to each other, and when I^P^ > S^P^, newly initiated origins, released from sequestration, compete with origins not yet initiated. Consequently, some origins are re-initiated while others are not initiated at all, leading to loss of synchrony (Olsson et al., 2003; Skarstad and Løbner-Olesen, 2003). This is exemplified by *dam* mutants without a sequestration period that initiate throughout the cell cycle (Figure 2B; Boye and Løbner-Olesen, 1990; Lu et al., 1994). *seqA* mutants are also asynchronous but have a higher origin concentration, possibly because DnaA is increased, relative to *dam* mutants (Figure 2C; Campbell and Kleckner, 1990; von Freiesleben et al., 1994). Increased levels of Dam will, due to faster re-methylation rates, reduce S^P^ and when this becomes shorter than I^P^, asynchrony follows (Figure 2C; Boye and Løbner-Olesen, 1990; von Freiesleben et al., 2000a). Excess SeqA protein delays initiation, prolongs the sequestration period but does not affect synchrony (Figure 2D; Bach et al., 2003; Charbon et al., 2011). During sequestration the activity of DnaA is lowered by RIDA and DDAH. RIDA is presumably accelerated by generation of new replication forks at initiation and hence more DNA loaded β-clamps (Moolman et al., 2014). Similarly, DDAH is increased shortly after initiation when the *datA* locus is duplicated and together they ensure a post-initiation decrease in the DnaA^ATP^/DnaA^ADP^ ratio (Figure 1). RIDA (Δ*hda*) and to a lesser degree DDAH (Δ*datA*) deficient cells fail to lower the ratio of DnaA^ATP^/DnaA^ADP^ to prevent re-initiation following sequestration. This results in asynchrony and early initiation at a reduced cell mass (Figure 2E; Kitagawa et al., 1998; Fujimitsu et al., 2008; Kasho and Katayama, 2013). On the other hand, the *dnaN*_*G*157*C*_ mutant, which is more active in RIDA (*dnaN* encodes the β-clamp), or extra copies of *datA*, results in delayed initiation and, for *dnaN*_*G*157*C*_ cells, also produces asynchrony (Figures 2F,G; Morigen et al., 2001; Gon et al., 2006; Charbon et al., 2011; Johnsen et al., 2011). During sequestration, the overall level of free DnaA is reduced by titration (Hansen et al., 1991; Kitagawa et al., 1996, 1998; Ogawa et al., 2002) and by arrest of *de novo* DnaA synthesis (Campbell and Kleckner, 1990).

## Modulation of timing of replication initiation by DNA binding proteins

Several DNA binding proteins affect either the cell mass at initiation, the initiation synchrony, or both. These proteins either bind specifically to *oriC* to affect DnaA binding, non-specifically to DNA to alter *oriC* topology, or they bind sequences important for the nucleotide bound status of DnaA.

### Proteins that specifically interact with *oriC* prior to initiation

The most important protein to interact with *oriC* prior to initiation is DnaA. Mutations in DnaA that affect nucleotide binding, such as *dnaA46*, are presumably somewhat deficient in formation of DnaA multimers on *oriC*, which results in delayed initiation and a prolonged initiation period (Skarstad et al., 1988; Boye et al., 1996). As sequestration remains unchanged (I^P^ > S^P^), *dnaA46* cells are asynchronous (Figure 2F; Skarstad and Løbner-Olesen, 2003). Mutations in DnaA that affect DNA binding, but not nucleotide binding (e.g., *dnaA204*), lead to late but synchronous initiation (Figure 2G; Skarstad et al., 1988; Torheim et al., 2000). The ability to form DnaA^ATP^ filaments on *oriC* therefore seems of greater importance for initiation synchrony than a tight anchoring to DnaA binding sites.

Conflicting data exist on the role of Fis for timing of initiation. Binding Fis to *oriC in vitro* is reported to either inhibit initiation of replication by inducing conformational changes at *oriC* that prevent orisome formation (Wold et al., 1996; Ryan et al., 2002, 2004), or have no effect on initiation (Margulies and Kaguni, 1998). Cells with a mutated primary Fis binding in *oriC* (*oriC131*) have an origin concentration similar to wild-type (Figure 2H; Weigel et al., 2001; Riber et al., 2009; Flatten and Skarstad, 2013). Fis-deficient cells, on the other hand, have a lowered origin concentration (Flatten and Skarstad, 2013; Kasho et al., 2014), suggesting that initiation is delayed (Figure 2F). However, because Fis affects multiple cellular processes due to its involvement in DNA organization one should be careful in assessing its role in initiation solely based on the behavior of Fis-deficient cells. Both Fis deficiency or loss of its primary *oriC* binding site result in initiation asynchrony (Figures 2F,H; Riber et al., 2009; Flatten and Skarstad, 2013), indicating that these cells are deficient for proper orisome assembly and/or for preventing premature DNA unwinding. The role of IHF in replication timing is less controversial. An *oriC* mutant with a disrupted IHF binding site (*oriC132*) is somewhat deficient in orisome formation and has delayed but synchronous initiation (Figure 2G; Weigel et al., 2001; Skarstad and Løbner-Olesen, 2003; Riber et al., 2009). *ihf* mutant cells also initiate replication at an increased mass per origin consistent with a stimulatory role of IHF on initiation. Cells deficient in IHF are on the other hand asynchronous (Figure 2F; von Freiesleben et al., 2000b). This is in agreement with an additional role of IHF for DnaA^ATP^ generation at *DARS2* (see below).

A number of proteins negatively regulate initiation of replication *in vitro*. These include ArcA that binds to 13 mer AT rich repeats, to DnaA box R1 and to the IHF binding site in *oriC*, and IciA that binds to 13-mer AT-rich repeats in *oriC* (Hwang and Kornberg, 1990; Lee et al., 2001). The impact of ArcA and IciA on replication initiation *in vivo* is modest (Nystrom et al., 1996) or not known, respectively. The stationary-phase induced CspD protein binds ssDNA to inhibit replication initiation and elongation *in vitro*, whereas no *in vivo* data are available (Yamanaka et al., 2001). Upon association with Cnu and/or Hha, H-NS (see below) binds to a specific sequence in *oriC* that overlaps DnaA box R5 (Kim et al., 2005; Yun et al., 2012). Cells deficient in Cnu and/or Hha are, however, similar to wild-type (Kim et al., 2005). Finally, the protein Rob binds to a single site in *oriC in vitro*, but does not affect initiation *in vivo* (Skarstad et al., 1993).

### DNA binding proteins that affect topology of *oriC*

In *E. coli* the genomic DNA is mostly negatively supercoiled (Wang et al., 2013). Unconstrained supercoiling of *oriC* contributes to the ease of duplex opening and is determined by transcription (not covered here; for review see Magnan and Bates, 2015) along with the actions of topoisomerase I and DNA gyrase enzymes (Wu et al., 1988). Mutations in topoisomerase I, which removes negative supercoils, result in initiation at a slightly reduced mass while synchrony is maintained (Figure 2I; von Freiesleben and Rasmussen, 1992; Olsson et al., 2003). Conversely, temperature sensitive *gyrB* mutant cells, with moderately reduced negative superhelicity of the chromosome, enhance the temperature sensitivity of a *dnaA46* mutant (Filutowicz, 1980) and show delayed synchronous initiations (Figure 2G; von Freiesleben and Rasmussen, 1991; Usongo et al., 2013). This suggests that initiation is facilitated by an increase in negative superhelicity of the chromosome. However, *topA-gyr* mutations influence chromosome segregation, R-loop formation and possibly induce stable DNA replication independent of *oriC* (Usongo et al., 2013, 2016) making it difficult to assess the effect of large changes in overall supercoiling on replication initiation. *In vivo*, nucleoid-associated proteins (NAPs; Dillon and Dorman, 2010), such as IHF, Fis, H-NS, HU, and MukFEB constrain negative supercoils to condense the chromosome and could therefore affect initiation of chromosome replication (Badrinarayanan et al., 2015; Lal et al., 2016). H-NS deficient cells have an increased negative superhelicity of the genome (Mojica and Higgins, 1997; Hardy and Cozzarelli, 2005). Yet, genetic evidence suggests that loss of H-NS hampers initiation (Katayama et al., 1996), and H-NS deficient cells initiate replication in synchrony at an increased cell mass (Figure 2G; Kaidow et al., 1995; Atlung and Hansen, 2002). The HU protein can substitute for IHF in DnaA-mediated unwinding of *oriC in vitro* (Hwang and Kornberg, 1992) although their mechanisms of action differ (Ryan et al., 2002). *In vivo*, genetic evidence suggests that loss of HU stimulates initiation despite decreased negative supercoiling (Louarn et al., 1984). Loss of MukB, involved in condensation of the bacterial chromosome (Hiraga et al., 1989; Cui et al., 2008), results in reduced negative supercoiling (Weitao et al., 2000), but initiations remain synchronous (Weitao et al., 1999). It is not known whether MukB affects the initiation mass. Finally, the starvation-induced NAP, Dps, binds non-specifically to *oriC*, and interacts with the N-terminus of DnaA, inhibiting DNA unwinding *in vitro*. Loss of Dps does not result in loss of synchrony, but increases the cellular origin content somewhat (Chodavarapu et al., 2008). In summary, it seems that NAPs modulate replication initiation but that the effect is not solely mediated through an effect on DNA supercoiling.

## Getting ready for the next round of replication

At later cell cycle stages DnaA^ATP^ is regenerated for the next initiation to take place (Figure 1). *E. coli* can rejuvenate DnaA^ADP^ to DnaA^ATP^ in a process assisted by acidic phospholipids (Saxena et al., 2013) or at two non-coding chromosomal sites called *DARS1* and *DARS2* (Fujimitsu et al., 2009). *DARS1* and *DARS2* are located in each replichore halfway between *oriC* and *terC*, and are duplicated after the end of sequestration. Multiple DnaA^ADP^ molecules form complexes with *DARS* to facilitate release of ADP resulting in apo-DnaA, which will primarily rebind ATP as this is more abundant than ADP within the cell (Petersen and Møller, 2000).

*DARS1* is not known to be regulated by any proteins, whereas rejuvenation at the more efficient *DARS2* locus is dependent on binding of both IHF and Fis (Kasho et al., 2014). While Fis binds *DARS2* throughout the cell cycle, IHF provides cell cycle specificity to *DARS2* activity by only binding and activating *DARS2* immediately prior to initiation to ensure an increase in DnaA^ATP^ level (Fujimitsu et al., 2009; Kasho et al., 2014). Extra copies of *DARS1* or *DARS2* will increase the overall DnaA^ATP^ level, which results in early initiation (Figures 2E,I) and for *DARS2* also extends I^P^, thereby resembling RIDA deficiency (Figure 2E; Fujimitsu et al., 2009; Charbon et al., 2011). Deletion of *DARS1, DARS2*, or both reduces the ability to reactivate DnaA for new initiations in the following cell cycle and results in delayed initiation (Figures 2F,G; Fujimitsu et al., 2009; Kasho et al., 2014; Frimodt-Moller et al., 2015). Loss of *DARS2* also increases the relative duration of the initiation period, leading to initiation asynchrony (Figure 2F; Fujimitsu et al., 2009; Frimodt-Moller et al., 2015). This suggests that both *DARS1* and *DARS2* are important for coupling initiation to cell mass increase, whereas only the cell-cycle regulated *DARS2* is crucial for maintaining initiation synchrony.

## Concluding remarks

Overall, timing of chromosome replication in *E. coli* takes place at least at two levels. First, initiation of replication is tightly coupled to cell mass increase through accumulation of DnaA^ATP^. Second, synchrony of initiations within the single cell is not necessarily connected to initiation mass but results from each origin being simultaneously initiated only once per generation, with asynchrony originating from failure to obey this once-and-only-once rule. DnaA remains the only replication protein solely required for initiation at *oriC*, but additional proteins act on *oriC* and elsewhere to assist in coupling of replication to cell growth and synchrony. In particular IHF and Fis display complex functions, targeting several regulatory sites. IHF has a dual role on replication initiation, acting both positively (i.e., binding to *DARS2* and *oriC*) and negatively (i.e., binding to *datA*). Also, IHF binds *oriC* at the pre-initiation stage and interacts with *datA* and *DARS2* following initiation. Binding of IHF to these regions is suggested to be temporally regulated so that IHF binds to *oriC*, to *datA* and to *DARS2* in a successive manner during cell cycle progression (Kasho and Katayama, 2013; Kasho et al., 2014). *In vivo, ihf* mutants display an initiation-compromised phenotype, indicating that the overall role of IHF on initiation of replication appears positive.

For a long time, the contribution of Fis in initiation regulation has been questioned. Recent studies do, however, suggest an overall positive role of Fis in replication initiation (Flatten and Skarstad, 2013; Kasho et al., 2014), which likely results from ensuring ordered orisome formation by preventing premature IHF binding and DNA unwinding (Leonard and Grimwade, 2015) and from stimulating DnaA^ATP^ rejuvenation at *DARS2*. As the cellular Fis level depends on both growth- rate and phase, it could adjust chromosome replication to the bacterial growth rate through its activity on *DARS2* (Kasho et al., 2014).

## Author contributions

All authors listed, have made substantial, direct and intellectual contribution to the work, and approved it for publication.

## Funding

This research was part of the Center for Bacterial Stress Response and Persistence (BASP) funded by a grant from the Danish National Research Foundation (DNRF120) and by a grant from the Novo Nordisk Foundation.

### Conflict of interest statement

The authors declare that the research was conducted in the absence of any commercial or financial relationships that could be construed as a potential conflict of interest. The reviewer MM declared a past co-authorship with the author ALO to the handling Editor, who ensured that the process met the standards of a fair and objective review.
